# Reducing potassium deficiency by using sodium fertilisation

**DOI:** 10.1007/s44154-022-00070-1

**Published:** 2022-11-02

**Authors:** Sarah J. Thorne, Frans J. M. Maathuis

**Affiliations:** 1 Department of Biology, University of Sheffield, Sheffield, S10 2TN UK; 2 Department of Biology, University of York, York, YO1 5DD UK

**Keywords:** Deficiency, Fertiliser, Nutrient, Potassium, Salinity, Sodium

## Abstract

Potassium (K) is the most abundant cation in the vast majority of plants. It is required in large quantities which, in an agronomic context, typically necessitates application of K in the form of potash or other K fertilisers. Recently, the price of K fertiliser has risen dramatically, a situation that is paralleled by increasing K deficiency of soils around the globe. A potential solution to this problem is to reduce crop K fertiliser dependency by replacing it with sodium (Na) fertiliser which carries a much smaller price tag. In this paper we discuss the physiological roles of K and Na and the implications of Na fertilisation for crop cultivation and soil management. By using greenhouse growth assays we show distinct growth promotion after Na fertilisation in wheat, tomato, oilseed and sorghum. Our results also show that up to 60% of tissue K can be substituted by Na without growth penalty. Based on these data, simple economic models suggest that (part) replacement of K fertiliser with Na fertiliser leads to considerable savings.

## Introduction

The human population explosion requires an ever-increasing agricultural output. This takes place when demand for land is greater than ever, and agronomic space competes with that needed for recreation, urbanisation, commerce, traffic, and wildlife. The increased demand for crops is mainly met by repurposing land use exemplified in the large-scale destruction of rainforests for cultivation of livestock and crops.

An important factor that is essential to achieve and maintain high yield, and thus slows down the expansion of arable land, is the extensive use of mineral fertilisers that supply crops with the major nutrients such as nitrogen, phosphorous and potassium (Marschner, 2012). Potassium (K) typically makes up 1–5% of a plant’s biomass which means that large amounts of this mineral are removed (‘offtake’) from the soil during harvest (several hundred kg per ha for a crop like wheat or rice). This demand for K means that each year, over 50 million tonnes of potassium fertilisers are applied to agricultural fields around the world (FAO, 2022) a number that is expected to grow at annual rates of approximately 2.5%. The most common K fertiliser, ‘potash’, is predominantly produced by mining in the Northern hemisphere (Ciceri et al., 2015), where large deposits have been found making this resource virtually inexhaustible.

In spite of potash application, many soils suffer from K deficiency, an affliction that is increasingly common across the globe. For example, more than half of the Southern Australian wheat belt and three quarters of Chinese rice paddies have been described as lacking adequate potassium (Roemheld and Kirkby, 2010). Under-fertilisation of K is frequently reported in Asia (Dobermann et al., 1998; Sheldrick et al., 2003; Pathak et al., 2010; Timsina et al., 2013) and a negative K nutrient balance (i.e. offtake>input) of approximately 20 kg/ha exists in African agriculture (Sheldrick and Lingard, 2002).

A major contributor to K deficiency in arable soil is K fertiliser costs which can easily exceed $100 per ha. In recent years prices have soared because of a range of factors including higher input costs, supply disruptions and export restrictions in potash producers. The price for a tonne of the cheapest form of potash (KCl) is now (July 2022) around ~$900, and twice as high as the previous year (Baffes and Koh, 2022). The enormous surge in price of K (and other) fertilisers is bound to make soil K deficiency even more widespread, especially in developing countries where agriculture is often based on smallholding, and low fertiliser affordability is already rife.

One potential approach to limit the negative effects of soil K insufficiency is the use of sodium (Na) salts to (partially) replace K applications. The rationale for this notion is the fact that in hydrated form, Na and K are chemically and structurally very similar and therefore some of the roles that K plays in plant cells could be fulfilled by Na (Maathuis, 2013; Kronzucker et al., 2013). This notion is not new; it has long been known that natrophilic crops like sugar beet produce much higher yields when fertilised with Na and many papers attest to the beneficial response of plants to low levels of Na, particularly when K is lacking (for review see Subbarao et al., 2003; Kronzucker et al., 2013). For example, we showed that addition of 1 mM NaCl to the growth medium did enhance growth rates of *Arabidopsis thaliana,* wheat, and barley (Maathuis et al., 1996). Similar observations have been made in other laboratories using other species. Clearly then, Na can be beneficial and ‘nutritious’, stimulating growth and vigour, especially when ambient K is low or deficient. In turn, this suggests that part of K fertilisation can be replaced by Na fertilisation in the form of Na-rock-salt which typically contains around 98% sodium chloride (Titler, 2012). This has two major advantages: firstly, at $50–100/t when bought in bulk (e.g. Maxisalt, 2022), rock-salt only costs a fraction of K fertiliser (£700–900/t in July 2022). The mining and production of rock-salt is also considerably more sustainable than that of K fertiliser with a CO_2_ footprint of around < 0.07 kg/kg for rock-salt and around 0.10–0.18 kg/kg for K fertiliser (Carbon cloud 2022; Chen et al., 2015; Hoxha and Christensen, 2019; ICL, 2021). Other positive effects of Na fertilisation are improved taste of many crops including asparagus, barley, broccoli and beet and increased nutritional value of livestock fodder (Dougherty et al. 1995).

In this paper, we will argue that Na fertilisation could have a much wider remit than it currently has, both in terms to the number of crops that could benefit and in terms of geography, particularly in areas where K is lacking. We will discuss the theoretical and practical implications, and economic feasibility of (partially) replacing K fertiliser with rock salt. The theoretical considerations will be underpinned by an exploration of the biochemical and biophysical functions of K and Na while some of the practical approaches will be deliberated using data from small scale laboratory growth assays.

## The role of potassium in plants

The level of potassium in the earth’s crust is around 2.6% (Diatloff and Maathuis 2013). In soils, the majority of K is dehydrated and coordinated to the oxygen atoms of insoluble minerals and therefore not available to plants. Typical concentrations of plant-available K in the soil solution vary between 0.1 and 1 mM (Marschner, 2012). In most cases, plant growth is stimulated by additional K supply via application of K fertiliser.

The physiological functions of K in plants can be classified as biochemical and biophysical (Maathuis, 2009; Ahmad and Maathuis, 2014). For example, K is pivotal for the activation of many enzymes, including ones that are pivotal in fundamental pathways such as pyruvate kinase in glycolysis. In vitro studies showed that enzyme activation typically occurs in the presence of 50–80 mM K^+^ activity, a value that agrees well with those measured in the cytoplasm. Binding of K^+^ to enzymes is in its dehydrated form and via coordination with six oxygens that may derive from carboxyl, carbonyl, and hydroxyl groups, and from water molecules (Benito et al., 2014). This process is very selective for K^+^ and cannot, or only partially can, be carried out by other similar ions such as Na^+^ or Li^+^ (Vašák and Schnabl, 2016). A further function of K in the cytoplasm is the screening and neutralising of excess negative charges that can be found on many macromolecules (Maathuis, 2009). In comparison with Na, K is less chaotropic and hence causes water molecules to favourably interact, which in turn stabilises intramolecular interactions of macromolecules such as proteins.

The largest fraction of tissue K is found in the vacuoles (Maathuis and Sanders, 1995; Walker et al., 1996). These organelles typically have K concentrations between 50 and 250 mM and easily contain 80–90% of total K. Vacuolar K is one of the most important generators of turgor and therefore is a critical factor in driving growth in many plants. The dominant role of K in turgor provision and water homeostasis is evident in processes such as pressure driven solute transport in the xylem and phloem (Benlloch-Gonzales et al., 2010), high levels of vacuolar K accumulation and the large fluxes of K^+^ that mediate plant movement such as stomatal aperture changes and nyctinasty of flower petals (Mano and Hasebe, 2021).

The biochemical functions of K mean that cytosolic K is under close homeostatic control in plant cells with ‘set point’ values of 100 to 150 mM (Maathuis and Amtmann, 1999; Walker et al., 1996). Non cytoplastic locations like the vacuole show a higher degree of variation where K levels are concerned. These numbers illustrate that plant tissues often contain 1–2% (DW) K which has to be derived from the soil. The uptake of K at the root-soil interface is catalysed by membrane located transporters with varying substrate affinities (Ahmad and Maathuis, 2014; Nieves-Cordones et al., 2014; Santa-Maria et al., 2000; Sustr et al., 2019). Passive uptake systems with low affinity consist of ion channels such as AKT1 in Arabidopsis (Ahmad and Maathuis, 2014; Nieves-Cordones et al., 2014; Sustr et al., 2019). Recent studies have revealed details of the signalling cascades that regulate AKT1 activity in response to K deficiency (Wang et al., 2018). High affinity K^+^ uptake is mediated by members of the HAK/KUP family (Rubio et al., 2008; Ahmad and Maathuis, 2014; Nieves-Cordones et al., 2014; Sustr et al., 2019. These are energised by the trans membrane proton (H^+^) gradient and as such are responsible for active K^+^ uptake. Intracellular partitioning, especially into the vacuole, is a function of K accumulation via members of the NHX (Na-H-exchangers) family, and vacuolar K release via K selective ion channels such as TPK1 (Gobert et al., 2007) and non-selective ion channels such as TPC1 (Peiter et al., 2005). Transporters that participate in long distance K^+^ transport have also been identified such as SKOR type channels that deliver K^+^ to the xylem for potassium translocation from root to shoot, while other channels are involved in phloem-mediated shoot to root K^+^ flux (Gaymard et al., 1998). The resulting cycling of K^+^ is believed to be important in overall K^+^ homeostasis and for the charge balancing of long distance transport of other (anionic) nutrients like nitrate on their way to the shoot.

## The role of sodium in plants

At around 2.8%, sodium (Na) makes up a comparable fraction of the earth’s crust as K (Diatloff and Maathuis 2013; Benito et al., 2014). Sodium is also abundant in the earth’s seas and oceans where its concentration is around 470 mM (approximately 5% w/w). Furthermore, it is found in almost all surface and subterranean freshwater bodies albeit in much lower concentrations (0.1 to 1 mM). With this abundance, it is hardly surprising then that most plants will get in contact with Na at some stage during their life cycle although the encountered levels may vary greatly (Subbaroa et al., 2003).

Although less true for aquatic species, the vast majority of land plants do not require Na for either growth and development or for reproduction (Maathuis et al., 1996; Kronzucker et al., 2013; Maathuis, 2013). An exception is a subgroup of C4 plants for which Na has been shown to be essential. This group contains examples such as Atriplex spp., *Kochia childsii*, millet, and a number of other C4 grasses. These C4 species require Na at trace levels to drive a particular process, namely the uptake of pyruvate into chloroplasts; the uphill transport of pyruvate is conducted by a Na^+^-pyruvate cotransporter that uses a transmembrane sodium gradient across the chloroplast envelope as energy source (Furumoto et al., 2011). In all other plants this function is mediated by a H^+^ coupled pyruvate carrier (Rao et al., 2016).

Another ‘biochemical’ role of Na has been suggested in the form of driving force to fuel high affinity K uptake. This idea came to fruition based on the discovery of particular K transporters from the HKT family. Initial studies in heterologous expression systems suggested that HKTs carried out coupled transport where (active) uptake of K^+^ was achieved by concomitant (passive) entry of Na^+^ with a 1:1 stoichiometry (Rubio et al., 1995; Jabnoune et al., 2009). However, a survey of several plants (Maathuis et al., 1996) showed that the physiological relevance of such a mechanism is likely to be minimal in terrestrial plant. In contrast, the same study did provide evidence that Na^+^-coupled high affinity K^+^ uptake is relevant in aquatic angiosperms.

Outside of the small group of C4 species mentioned above there are no known plants for which Na can be classified as an ‘essential nutrient’ (Maathuis, 2009) and the above suggests its capacity to substitute biochemical roles of K is limited (Benito et al., 2014). In fact, concentrations of Na at levels comparable to those found for K (~ 100 mM) can readily induce toxicity symptoms in cellular compartments such as the cytosol. This may be due to substitution of K by Na in enzyme cofactors, resulting in compromised protein functioning (e.g. Flowers et al., 2015). Compared to K, Na is also considerably more chaotropic; It tends to disrupt water structure such as the hydrogen bonds between water molecules and polar groups of proteins, and therefore can interfere with their biochemical activity (Benito et al., 2014; Isayenkov and Maathuis, 2018). This implies that the beneficial properties of Na are mostly found in replacing biophysical functions of K and in some literature it is hence termed a ‘functional nutrient’ (Subbaroa et al., 2003; Kronzucker et al., 2013; Maathuis, 2013). An example of benefits is the accumulation of Na in the vacuole where it provides turgor and as such can positively affect plant growth, without detrimental impact on sensitive biochemical and metabolic processes.

Several pathways have been shown to be present in plant roots that allow Na to enter the symplast through ion channels and carrier type transporters. Ion channels that are relevant in this respect are members of the glutamate like receptor (GLR) family (Tester and Davenport, 2003), cyclic nucleotide gated channels (CNGCs; Gobert et al., 2006), and probably include further, as yet unknown, non-selective cation channels (Demidchik and Maathuis, 2007). In addition, Na^+^ uptake can be mediated by carrier type transporters of the high affinity potassium transporters (HKTs) (Haro et al., 2010; Mian et al., 2011; Rodriguez-Navarro and Rubio, 2006). The latter family contains HKT isoforms that either transport Na^+^ as such (subfamily 1), with a range of affinities, or cotransport Na^+^ and K^+^ (subfamily 2) and thus are an example where K^+^ and Na^+^ uptake interfere with each other. The two subfamilies have slightly different amino acid sequences in their pore and hence substrate selectivities.

Non selective ion channels are likely to mediate a considerable Na influx in saline conditions (e.g. Maathuis and Sanders, 2001; Demidchik and Tester, 2002; Demidchik and Maathuis, 2007; Zhang et al., 2010) but less so at the much lower Na concentrations that prevail in the context of Na fertilisation. In that case specific members of the HKT family with intermediate affinity could be active. For example, an HKT from *Physcomitrella patens* was identified by Haro et al. (2010) with a Km for Na of around 1.5 mM whereas in barley root cortical cells HKT2;1 is involved in Na uptake with a Km of around 6 mM (Mian et al., 2011). Similar mechanisms have been identified in other grasses (Haro et al., 2010). In many species it has been shown that high affinity Na^+^ uptake is induced when K^+^ becomes deficient. This is partly due to transcriptional regulation but can also have a mechanistic basis in the transport protein itself; in barley, HKT2;1 (Mian et al. 2011) mediates Na^+^ transport but this function is distinctly inhibited whenever millimolar levels of K^+^ are present. In addition, the study by Haro et al. (2010) on high affinity Na^+^ uptake in plants also strongly suggested there is another class of transporters involved that is fundamentally different from HKT transporters but has as yet not been identified.

Once Na has crossed the plasma membrane, a large proportion will be sequestered in the vacuole where Na can substitute K in its role as osmoticum to generate turgor. Vacuolar Na accumulation is carried out by members of the NHX (Na-H-exchanger) family, energised by the trans-tonoplast proton motive force (e.g. Jiang et al., 2010).

For Na to be an effective substitute for K, it will need to be distributed in all major organs which requires long distance transport, especially from root to shoot. Similar to K, Na can counterbalance the negative charge of anionic nutrients such as nitrate in the xylem (Alvarez-Aragon and Rodriguez-Navarro, 2017). Loading of Na into the xylem is partially facilitated by members of the NHX family, such as AtNHX7 (SOS1; Zhang et al., 2010) in Arabidopsis. Cation-proton exchangers (CHXs) may also be involved while HKTs have been shown to be crucial in limiting the Na load to the shoot (e.g. Haro et al., 2010; Zhang et al., 2010) through reabsorbing Na from the xylem sap. The latter is unlikely to be particularly relevant in the context of Na fertilisation.

## Examples of beneficial sodium application in crop species

**Fig. 1:**
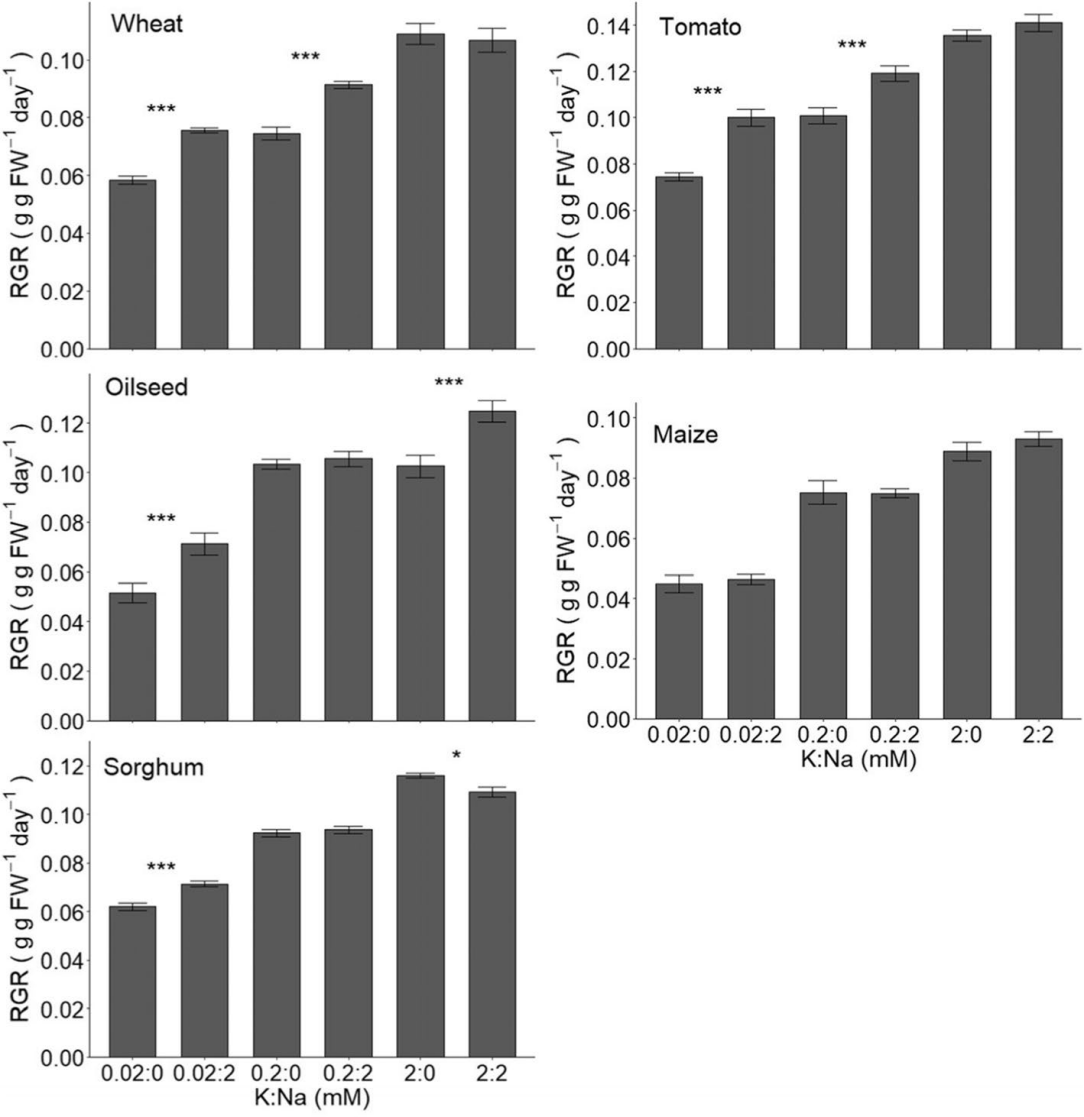
Relative growth rate (RGR) after 4 weeks of treatment with varying levels of K in the presence or absence of Na for wheat, tomato, oilseed, maize, and sorghum. Shown are the means ± SE of six replicates. Stars show significant differences between treatments with and without Na at each level of K as determined using t-tests. * *p* < 0.05, *** *p* < 0.001

**Fig. 2:**
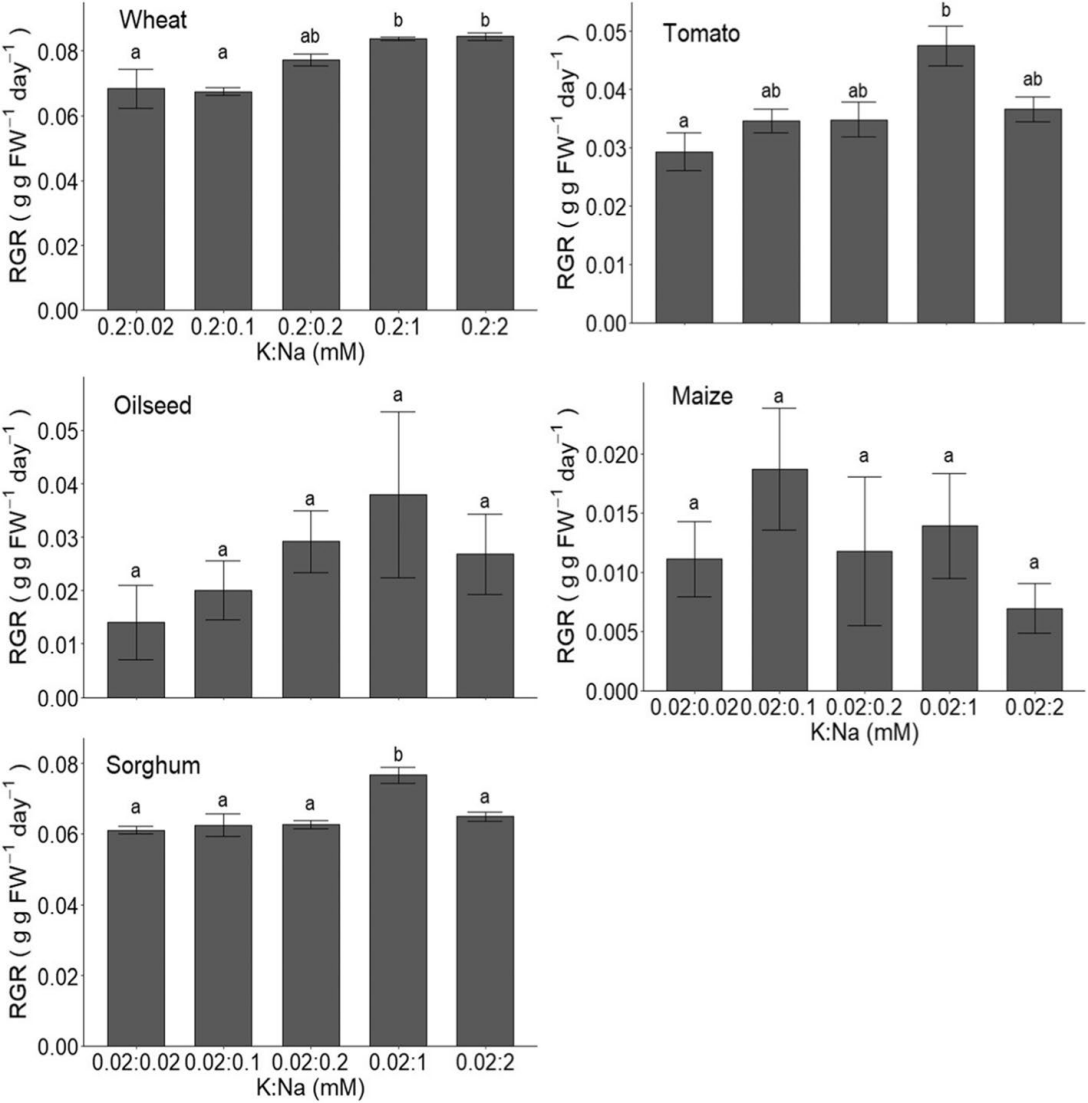
Relative growth rate (RGR) after treatment with varying levels of Na for wheat, tomato, oilseed, maize, and sorghum. Plants were harvested after 6 weeks of treatment (4 weeks for oilseed). Plants were grown with 0.02 mM K (0.2 mM for wheat). Shown are the means ± SE of three replicates. Different letters represent statistically different results (*p* < 0.05) between treatments as determined by one-way ANOVA followed by Tukey’s *post-hoc* test

From the above sections it can be concluded that, at least in principle, a large fraction of tissue K can be substituted by Na without negative consequences. This particularly pertains to biophysical roles such as accumulation in vacuoles to generate turgor but is also likely to include charge balancing of long distance transport in the xylem (e.g. Subbaroa et al., 1999; Subbarao et al., 2003). To generate a consistent set of data for some important crop species we carried out growth assays on five widely grown staples. Figures 1 and 2 show growth responses using hydroponic cultivation on a range of K:Na ratios. Included are three C3 species (wheat, oilseed rape and tomato), and two C4 species (maize and sorghum) of which the latter is known to show high tolerance to salinity and drought. Figure 1, where the [K] varies between 0.02 and 2 mM and [Na] is 0 or 2 mM, shows that Na promotes relative growth rates (RGRs) in four out of the five tested crops. No effect of Na application was found for maize. Although other studies did not find growth promotion in sorghum (e.g. Subbaroa et al., 2003) we observed around 15% increase in growth when ambient K was 0.02 mM and Na was added. Interestingly, a small negative effect of Na treatment was recorded in the presence of 2 mM K. Proportionately, the measured positive responses were generally greater when ambient K levels are low. This is clearly illustrated in sorghum but also in wheat where the growth advantages after Na addition are greatest when K is 0.02 mM, intermediate when it is 0.2 mM and absent in the presence of sufficient K (2 mM). To gain further insight into the optimal concentration of Na, the [K] was fixed and [Na] varied between 0.02 and 2 mM (Fig. 2). This showed that the optimum [Na] is typically around 1 mM and remarkably similar for those species that showed improved growth.

**Fig. 3:**
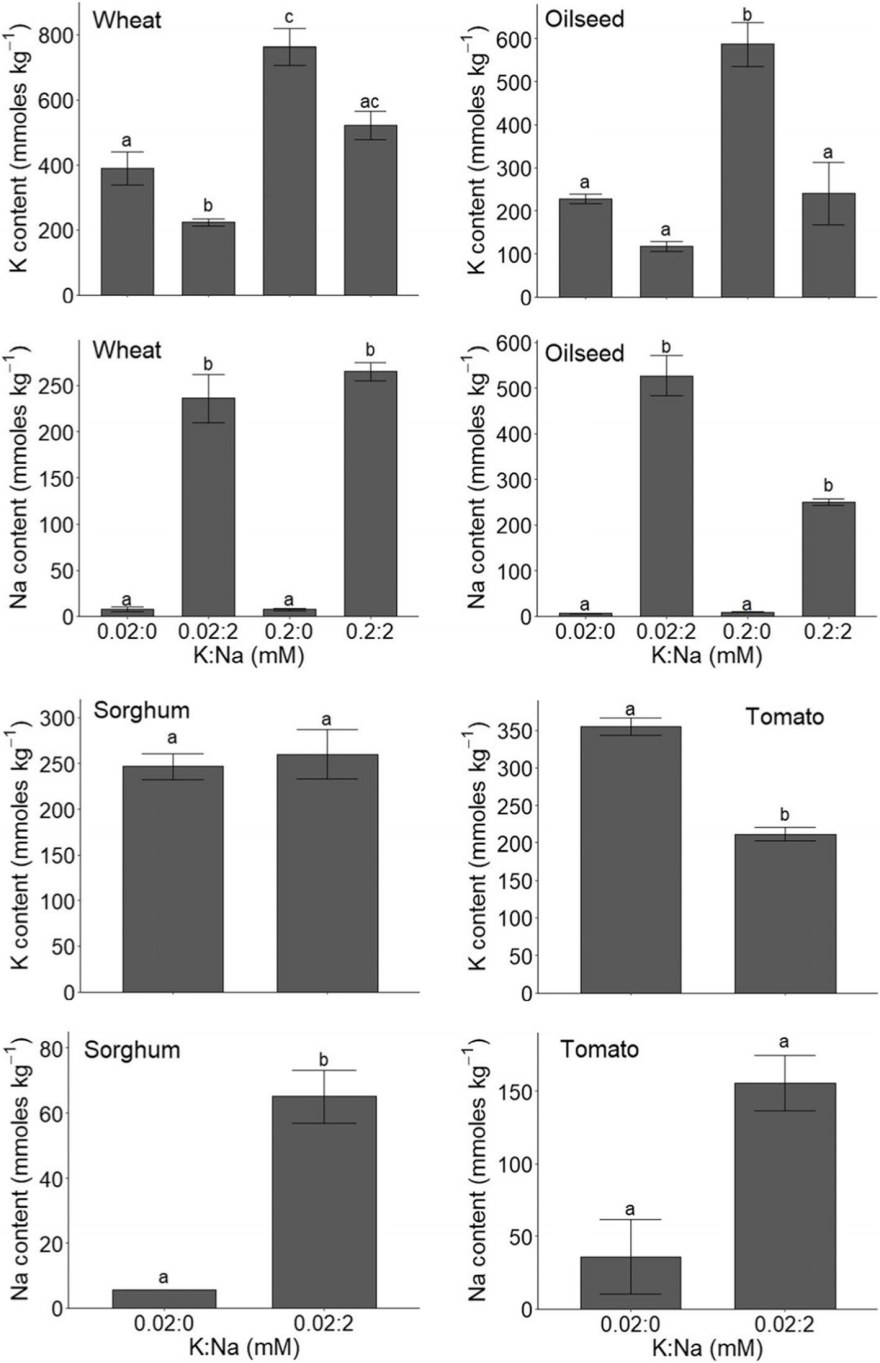
Tissue K and Na content of wheat, oilseed, sorghum, and tomato grown at different levels of K and Na. Shown are the means ± SE of three biological replicates per species. Different letters represent statistically different results (*p* < 0.05) between treatments as determined by two-way ANOVA followed by Tukey’s *post-hoc* test

Figure 3 depicts the tissue K and Na contents of the plants used for Figs. 1 and 2. There are large differences between conditions and species. For example, in oilseed more than 50% of tissue K is lost when plants are Na fertilised which is paralleled by an increase in growth of around 45% (at 0.02 [K], see fig. 1). When [K] in the medium is 0.2 mM we did not observe a growth advantage (Fig. 1) but there is still a large and similar reduction in tissue K and thus a potential saving in K fertiliser costs. On the other hand, in sorghum only a small fraction (~ 15%) of tissue K gets substituted by Na. These data emphasise the need for detailed information regarding biomass production and tissue ion content for different species, using varying K and Na levels.

## Sustainability and the risk of causing salinisation

Soil salinisation can arise through natural causes, such as the local geology or proximity to coastal areas, or be man-made, for example through the use of irrigation water that contains high concentrations of salts, through excessive water extraction, or in a wider context, through CO_2_ emissions that lead to hotter climates. Human activities have led to encroaching salinisation in many regions of the world and saline soils are now a global menace for agriculture. The latter is the case because, apart from a few halophytes like *Salicornia* (samphire), crops tend to be salt sensitive (e.g. Munns and Tester, 2008). Naturally, any further significant Na load on arable land has to be prevented which means Na fertilisation has to be carefully managed and monitored. To be sustainable, a balance between input and removal of Na is necessary in order to maintain (low) salt levels. Input of Na fertiliser is largely under control of the farmer whereas Na removal is made up out of Na offtake and Na leaching. Offtake can easily be determined by analysing tissue samples and applying those to total yield. The third factor, leaching, is the least predictable and will depend on many factors such as soil cation exchange capacity, soil compaction and drainage, precipitation, etc. Nevertheless, measuring soil conductivity is straight forward and should give an excellent estimate of total soil salinity. If necessary, this can be expanded with flame photometry, atomic absorption spectrophotometry or inductively coupled plasma techniques to determine cation composition, although this will clearly not be available to most farmers. Careful monitoring of these three factors should prevent salt accumulation and thus prevent long term soil degradation such as salt induced soil compaction and associated yield penalties.

## Economic feasibility

Although field data are lacking, the above examples and data found in the literature allow us to make some cost-benefit estimates regarding the application of rock-salt as a sodium source to substitute tissue K. For cereals like wheat and rice, a 10 t per ha grain yield is a realistic number. This will be accompanied by around 3 t of straw (Roth et al., 2021). Assuming both grain and straw are removed, and K content is 2% on average (e.g. Krishnasamy et al., 2014), harvesting would generate an offtake of 260 kg per ha. The K content of potash is normally ~ 60% which, assuming bulk prices of $900 per tonne, translates into a price-tag of $1500 per tonne K. Offtake replenishment of 260 kg K therefore carries a cost of $390 per ha. Figure 3 suggests that around one third of K is replaced by Na. Rock-salt costing $75 per tonne contains around 40% Na, giving a price of $190 per tonne Na. Thus, if a third of K is replaced by Na it would lead to a saving of $((260*0.33)*1.5)-((260*0.33)*0.19) = 113 per cultivated ha. For oilseed the potential ‘replacement fraction’ may be as big as 60% (Fig. 3) and thus would generate greater savings of $((260*0.6)*1.5)-((260*0.6)*0.19) = 204 per ha (assuming similar yield tonnage and tissue K content of 2%).

The above calculations are based on the assumption that all supplied fertiliser (potash or rock-salt) is taken up by the plants (100% efficiency). Clearly this is not realistic since typical potash use efficiencies are only 40–50% (Shin, 2014). We did not find any use efficiency value for rock-salt in the literature but if it is comparable to that for potash the above input numbers simply need a generic scaling factor (e.g. a value of 2, assuming 50% use efficiency).

A less than 100% efficiency also implies that a large amount of applied fertiliser remains in the soil. Some of this fraction will be washed out. Losses of K via leaching typically vary between 1 and 10% of total input depending on soil type (Alfaro et al., 2004; Lu et al., 2022). Na has a much higher charge density than K (Benito et al., 2014), which promotes binding to soil minerals and hence its leaching is likely to be lower than that of K (Black and Abdul-Hakim, 1984). In either case, fertiliser application rates should be adjusted to prevent long term accumulation. This is especially important in the case of Na because it can cause soil structure deterioration (Black and Abdul-Hakim, 1984).

Apart from the size of the portion of K that is substituted by Na it is important to assess what growth or yield advantage is achieved. Indeed, maximum economic gain will be made in conditions that (a) provide the largest reduction in K offtake and (b) that are paralleled by maximum growth advantage. [Other, more generic factors such as produce prices will also impact but for simplicity are not considered here.] To conceptualise growth advantage, it is more illustrative to express it as a proportion of growth under ‘optimum’ K conditions (2 mM). For example, the tomato data in Fig. 1 show that the 0.2:2 mM (K:Na) treatment reaches almost 90% of the maximum growth while the 0.02:2 mM (K:Na) condition yields around 75%. These figures pertain even though the Na-induced growth increase is ~ 15% when K is 0.02 mM while it is only ~ 11% at 0.2 mM K.

## Conclusions

Scientific literature regarding the nutritional aspects of Na in plants is still limited and this greatly constrains development of commercial applications such as Na-based fertilisers. Laboratory and greenhouse based reports are available that suggest various benefits from Na fertilisation. For example, Na is likely to positively affect taste and palatability of crops, especially in the case of leafy vegetables and where livestock fodder is concerned (Cushnahan et al., 1996; Subbarao et al., 1999). In addition, the high prices of K fertiliser provide a powerful economic incentive for the application of Na fertilisation. However, this will require extensive in situ surveys and currently there is a particular shortage of (long term) field studies. These will have to be conducted for multiple species, using a range of fertiliser K:Na ratios and cultivation on different types of soil. It is also imperative that such works include effects on yield since most literature is limited to data on biomass which does not necessarily correlate with agronomically important parameters such as grain yield and harvest index (Thorne et al., 2022). A further open question concerns the interaction of sodium fertiliser with nutrients other than K. At moderate NaCl concentration (20 mM) nitrate has been shown to enhance both Na uptake and its translocation to the plant shoot (Álvarez-Aragón and Rodríguez-Navarro, 2017). Other work showed the reciprocal effect; a stimulation of root nitrate uptake after addition of NaCl (Liu et al., 2021).

What is clear is that the capacity of Na to replace K varies as a function of ambient K:Na and also varies between crops and genotypes within crop species (Figs. 1, 2 and 3; Kronzucker et al., 2013; Subbaroa et al., 2003). For example, great genotypic variability has been observed in tomato (Subbaroa et al., 2003) providing an important role of selective breeding for ‘high’ Na produce. This variation is likely to depend on processes that drive Na homeostasis such as partitioning between cytoplasm and vacuole, critical Na concentrations and partitioning between tissues and organs, processes that are only partially understood (Isayenkov and Maathuis, 2018).

## Methods

For hydroponics experiments, plants were germinated in sand for 1 week and subsequently transferred to a modified Hoagland’s solution which comprised 0.5 mM MgSO_4_, 3 mM Ca (NO_3_)_2_, 1 mM NH_4_H_2_PO_4_, 4.6 μM MnCl_2_, 23 μM H_3_BO_3_, 0.05 μM (NH_4_)6.Mo_7_O_24_, 0.4 μM ZnSO_4_, 0.3 CuSO_4_, and 33 μM FeCl_3_ with the pH adjusted to 5.7–6 using 1 M methyl-glucamine.

For data presented in fig. 1, six replicate plants per species were grown in 0.02 mM, 0.2 mM, or 2 mM KCl with or without 2 mM NaCl. For data presented in fig. 2, three replicate per species plants were grown in 0.02, 0.1, 0.2, 1, or 2 mM NaCl with 0.02 mM KCl (0.2 mM KCl for wheat). Plants were grown under controlled glasshouse conditions (16 h daylight; 20 °C /15 °C day/night). Plants were weighed prior to the start of treatment and after 4 weeks (fig. 1) or 6 weeks (fig. 2) of treatment. Relative growth rate (RGR) was calculated as:

$$RGR\ \left(g\ g\ {FW}^{-1}\ {day}^{-1}\right)=\frac{\ln \left( final\ weight\right)-\ln \left( initial\ weight\right)}{time\ (days)}$$

Statistical analyses were performed using R software (version 3.6.1, R Core Team, 2020). Summary statistics were calculated using the Rmisc package (Hope, 2013) and graphs were produced using the ggplot2 package (Wickham, 2016). Two-way analysis of variance (ANOVA) was used to test the effect of Na and K level on RGR and cation content. Data normality was checked using Shapiro-Wilk tests and homogeneity of variance was tested using Levene’s tests. Both RGR and cation values were log transformed to satisfy the test assumptions. A significance level of *P* < 0.05 was used for all analyses. Significant results were analysed by performing Tukey’s Honest Significance Difference (HSD) post-hoc tests using the emmeans package (Lenth, 2021).

## Data Availability

N/A
